# Juvenile polyposis without a germline variant in *SMAD4/BMPR1A*: defining a clinically distinct polyposis syndrome

**DOI:** 10.18632/oncotarget.27999

**Published:** 2021-08-31

**Authors:** Suzanne P. MacFarland, Bryson W. Katona

**Keywords:** juvenile polyposis syndrome, SMAD4, BMPR1A, polyps

Juvenile Polyposis Syndrome (JPS) is a rare gastrointestinal (GI) polyposis syndrome in which approximately half of cases are caused by constitutional changes in *SMAD4* or *BMPR1A*. The other half of JPS cases lack a known genetic driver (i.e., mutation-negative) and are instead diagnosed clinically based on existing criteria including having five or greater pathologically defined juvenile polyps in the lower GI tract, juvenile polyps in the upper and lower GI tract, or any number of juvenile polyps with a family history of JPS. JPS is associated with increased risk of gastrointestinal cancer (between 9–50% lifetime risk, with median age of onset 34 years) [1–5]. Consequently, individuals with JPS require regular, lifelong screening with colonoscopy and upper endoscopy, and may also require colectomy or gastrectomy for high burden of disease or to treat malignancy. It has been previously noted that patients carrying *SMAD4* variants have a higher risk of upper GI disease, including increased gastric cancer risk, and these individuals also have risk of hereditary hemorrhagic telangiectasia [6]. However, there remained very limited literature comparing the phenotypic differences between JPS patients with and without a mutation in *SMAD4* or *BMPR1A*.

**Figure 1:**
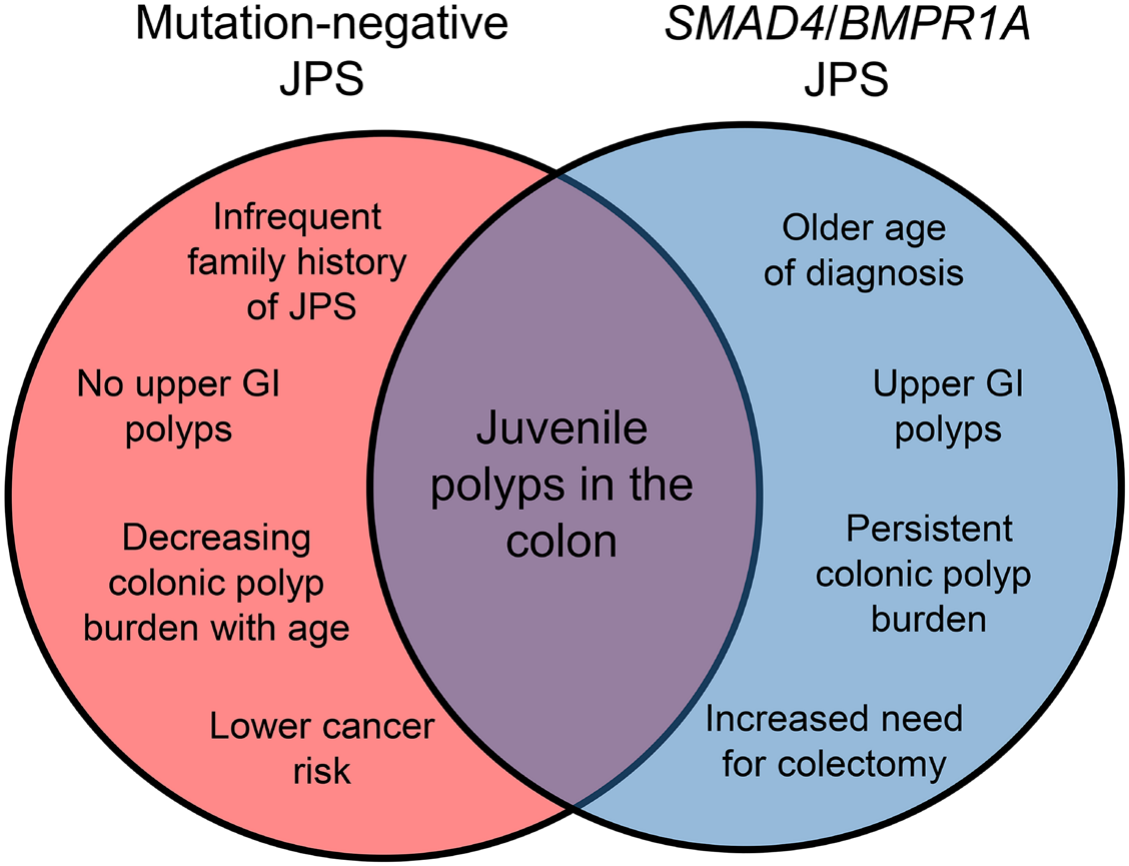
Overview of phenotypic differences between mutation-negative JPS and JPS due to a germline variant in *SMAD4/BMPR1A*.

To address this important unanswered question, we recently reported on a large multi-institutional JPS cohort, comparing patients with JPS due to a mutation in *SMAD4* or *BMPR1A* (*n* = 54) to those without an identifiable mutation (*n* = 64), with significant phenotypic differences noted (Figure 1) [7]. These include differences in presentation, with mutation-negative patients being diagnosed at a younger age (median age 5 years vs. 18 years) and had last follow-up at a younger age (11 years vs. 33.5 years), and were less likely to have a family history of JPS (6% vs. 52%). When separated by pediatric versus adult institution, the cohort from pediatric institutions had a much lower rate of mutation-positive patients (22% from pediatric institutions vs. 83% from adult institutions). Additionally, we also found that mutation-negative patients had no reported upper GI polyps, as compared with 57% of those individuals with JPS who had a *SMAD4* or *BMPR1A* mutation. Patients without a mutation were also much less likely to require a colectomy (3.1% vs. 33.3%). Finally, while the difference was not statistically significant given the age difference in the populations, those without a mutation in *SMAD4* or *BMPR1A* had a lower incidence of gastrointestinal cancer (1.6% vs. 18.5%). The data set was limited in its retrospective nature and had limited data on mutation-negative patients in adulthood; however, to date this is the largest data set of its kind to define differences in individuals with JPS both with or without a *SMAD4* or *BMPR1A* mutation.

The differences observed in our multi-institutional JPS cohort show that mutation-negative JPS has a distinct phenotype compared to JPS with a mutation in *SMAD4/BMPR1A*. These differences are relevant for informing the clinical management of mutation-negative JPS patients, an area that previously lacked data to permit differing clinical recommendations. Consequently, our data were incorporated into the most recent National Comprehensive Cancer Network (NCCN) surveillance guidelines, which now separate mutation-negative JPS from JPS due to a *SMAD4/BMPR1A* mutation to allow for relaxed GI surveillance in mutation-negative JPS [8]. Specifically, the NCCN now recommends lengthening the GI surveillance interval for mutation-negative JPS to 5 years if no polyps are identified, whereas surveillance should be continued every 1–3 years in those with a *SMAD4/BMPR1A* variant [8]. Further consideration might be given to even further limiting upper GI surveillance in mutation-negative JPS given the limited upper GI polyp burden identified in these individuals. However, further longitudinal evaluation of the mutation-negative cohort will be needed to gather the necessary data to support this recommendation. Appropriate management of a cancer risk syndrome has lifelong implications and can cause significant stress, especially when diagnosed at a young age. Therefore, the ability to risk-stratify based on genotype can be helpful both for families and providers, limiting the need for unnecessary endoscopic procedures in patients for whom overall risk is low.

Additionally, the difference in heritability and polyp burden that our data highlighted may also suggest a difference in underlying disease pathogenesis in individuals without a detectable germline variant. For example, given the younger age of diagnosis and limited family history within this mutation-negative cohort, a lower-penetrance germline variant might be considered, or an autosomal recessive pattern of inheritance. Further, somatic mosaicism or epigenetic alterations might also drive the sporadic JPS phenotype. Further research is needed to better understand the cause of clinical JPS in patients who do not have a mutation in *SMAD4* or *BMPR1A*.

In conclusion, our multi-center study is the largest study to date investigating the differences between individuals with or without a known genetic cause of JPS. Clear differences including younger age of presentation, lower chance of heritability, and lower burden of disease (especially upper GI disease) all suggest that patients without a mutation in *SMAD4* or *BMPR1A* have a distinct clinical phenotype that may potentially be viewed as a unique polyposis syndrome compared to JPS due to a *SMAD4/BMPR1A* variant. Although individuals with mutation-negative JPS still need to be followed closely, they may ultimately have lower overall cancer risk.
